# Whole tumor transcriptome: a possibility for diagnosis, prognosis, and therapy in precision oncology using computational analysis

**DOI:** 10.3389/fmed.2026.1759083

**Published:** 2026-01-20

**Authors:** Ian Jhemes Oliveira Sousa, Kerolayne de Melo Nogueira, Ester Miranda Pereira

**Affiliations:** 1 Northeast Biotechnology Network, Federal University of Piauí, Teresina, Brazil; 2 Postgraduate Program in Pharmacology, Universidade Federal do Ceara, Fortaleza, Brazil; 3 Laboratory of Immunogenetics and Molecular Biology, Universidade Federal do Piaui, Teresina, Brazil

**Keywords:** computational biology, personalized therapy, precision oncology, RNA-Seq, tumor transcriptome

## 1 Introduction: from morphology to functional precision in oncology

Modern oncology has witnessed a paradigm shift, moving from therapeutic approaches based on histopathological and anatomical characteristics to personalized strategies defined as Precision Medicine (1). This evolution is driven by the increasing ability to characterize tumors molecularly, identifying the genetic and epigenetic alterations that drive disease growth and progression. Although DNA sequencing (genomics) has provided crucial insights into cancer-associated mutations, a comprehensive understanding of tumor biology increasingly depends on functional molecular analyses, particularly transcriptome profiling (2).

The transcriptome, which represents the complete set of RNA molecules in a cell at a given time, reflects real-time genetic activity and, consequently, the functional state of a tumor (1). Although most advances in transcriptomics and proteomics are associated with the study of cancer, these approaches have also been widely applied to elucidate pathophysiological mechanisms in other complex diseases. Investigations in this direction demonstrate how the characterization of molecular profiles can reveal crucial early events in pathogenesis, providing support for the development of more precise diagnostic and therapeutic strategies (3).

High-throughput RNA sequencing (RNA-seq) technologies have enabled an unprecedented exploration of this molecular universe, revealing not only the expression of protein-coding genes, but also non-coding RNAs, splicing isoforms and gene fusions, which play critical roles in oncogenesis and treatment response (2). Total tumor transcriptome analysis, therefore, offers a comprehensive and functional view of the molecular identity of a cancer, overcoming the limitations of static genomic analysis by capturing the dynamics of cellular adaptation. This integration makes it possible not only to identify molecular signatures with diagnostic and prognostic value, but also to delineate more specific therapeutic targets, consolidating itself as an essential element for the development of the next generation of personalized cancer strategies (4).

Extracting biologically relevant patterns from these high-dimensional datasets will enable the discovery of more sensitive and specific biomarkers, the refinement of patient stratification processes, and the design of more effective and personalized therapeutic interventions. Thus, the most immediate impact of transcriptomics lies in its transformative potential on cancer diagnosis (5). While conventional histopathology, although fundamental, has limitations in capturing the complexity and heterogeneity of tumor behavior, transcriptome analysis paves the way for more refined molecular characterization (6).

## 2 Clinical applications of whole tumor transcriptomics in precision oncology

This potential extends to early detection, where the analysis of cell-free RNA (cfRNA) in liquid biopsies can allow the identification of tumors even before the onset of clinical symptoms (7). Specific transcriptomic signatures of cfRNA can indicate the presence of tumor cells and their tissue of origin, facilitating faster interventions. In addition, many cancers, such as breast and lung cancer, are collections of diseases with distinct molecular profiles. The transcriptome allows the identification of molecular subtypes, such as Luminal A, Luminal B, HER2-enriched, and basal subtypes in breast cancer, that are not discernible by histopathology but are essential to guide treatment selection (8).

This molecular precision is also invaluable for cancers of unknown primary, which account for 3%−5% of all cancer cases. For these challenging diagnoses, metastasis transcriptome analysis can reveal the tumor's tissue of origin, allowing for more targeted and effective therapy (9). However, the complexity of RNA-seq data requires sophisticated computational methods, such as machine learning algorithms like Support Vector Machines and neural networks, to build accurate classifiers (10). At this stage, it is essential to clearly distinguish exploratory models that demonstrate strong performance in experimental cohorts from computational tools that have achieved clinical validation and meet regulatory requirements for routine diagnostic use.

Beyond this distinction, important challenges persist, including the confounding effects of tumor heterogeneity, where the presence of non-cancerous cells can obscure tumor-specific signals (11). Furthermore, variations in laboratory protocols require robust data normalization methods to ensure comparability and reproducibility (12). In addition to refining diagnosis, the transcriptome provides an equally powerful lens for predicting disease trajectory. Prognosis, which underpins decisions about treatment intensity and follow-up, is traditionally based on clinicopathological factors that often fail to capture the biological aggressiveness of a tumor. Transcriptomic analysis can identify gene expression signatures that stratify patients into high- and low-risk groups for recurrence or disease progression, thus personalizing treatment intensity and avoiding overtreatment or undertreatment (13).

This is particularly critical for predicting cancer recurrence, a major challenge in oncology. Transcriptomic signatures can predict the likelihood of recurrence, allowing for more rigorous monitoring and the proactive use of adjuvant therapies in high-risk patients (14). In fact, this concept is already in clinical practice with tests such as Oncotype DX^®^ and Prosigna^®^ for breast cancer, which use gene expression signatures to predict the risk of recurrence and guide chemotherapy decisions (15).

The computational approaches behind these tools involve machine learning algorithms trained on large patient cohorts. More advanced methods, such as gene co-expression network analysis, provide additional prognostic insights by identifying co-regulated gene modules linked to specific biological processes, such as cell proliferation or invasion (16). Integrating this molecular data with established clinical factors in multivariate models can further increase prognostic accuracy (17).

However, the development and validation of these models require large, well-annotated patient cohorts with long-term follow-up, and any model must be externally validated to ensure its generalizability. Furthermore, improving the interpretability of these often “black box” computational models is crucial for building clinical confidence and understanding the underlying biology. Ultimately, improved diagnoses and prognoses should translate into more effective treatments, which is the central promise of Precision Medicine. The whole tumor transcriptome is fundamental in this endeavor, providing detailed information to guide the selection of therapies that maximize efficacy and minimize toxicity (18).

Its most direct application is the identification of actionable therapeutic targets. The transcriptome can reveal the overexpression of genes that encode drug targets, such as growth factor receptors, or identify unique molecular vulnerabilities, such as targetable gene fusions and aberrant splicing variants that are invisible to genomic analysis alone (19).

Furthermore, transcriptomic signatures can predict a tumor's sensitivity or resistance to specific treatments. For example, while PD-L1 protein expression is a common biomarker for immunotherapy, a broader transcriptomic profile of the tumor immune microenvironment can offer a more comprehensive prediction of response to PD-1 blockade (20).

This dynamic monitoring capability is also critical for tracking treatment resistance. As tumors evolve under therapeutic pressure, serial transcriptomic analysis, especially from non-invasive liquid biopsies, can detect the emergence of resistance mechanisms, allowing physicians to modify treatment strategies in real time (21).

## 3 Barriers, limitations, and future directions

Despite these advances, the translation of these vast transcriptomic data into clinical decisions is computationally hard, relying on pathway and network analyses to understand drug effects and on machine learning models trained on large drug response datasets to predict sensitivity *in silico* (22). The main challenges in this domain are the complex and multifactorial nature of drug resistance and the long and high risk of developing new drugs against newly identified targets. The path to the widespread clinical implementation of transcriptome-guided oncology requires overcoming several significant practical obstacles. The main one is the need for rigorous standardization of sample handling, sequencing, and data analysis protocols to ensure that results are reproducible and comparable across different centers (13, 23).

The enormous volume of data generated also requires a robust and accessible computational infrastructure for storage and processing, which remains a bottleneck for many institutions (24). To achieve a truly holistic understanding of cancer, it will be essential to integrate transcriptomic data with other “omics” layers such as: genomics, proteomics, and metabolomics, a task that represents a major computational and statistical challenge (25).

As we deal with this deeply personal patient data, we need to navigate the associated ethical and privacy issues, ensuring the existence of robust regulatory frameworks to protect patients while enabling research (26). However, the future is rich in opportunities as continuous advances in artificial intelligence and deep learning promise to uncover even more complex patterns in transcriptomic data, and technologies such as single-cell RNA sequencing (scRNA-seq) are already providing unprecedented results. Finally, standardization of analyses and the application of best practices remain fundamental to the consolidation of transcriptome-based precision oncology (27–29).

In conclusion, whole-tumor transcriptome analysis represents an indispensable cornerstone for the evolution of precision oncology. By capturing functional dynamics that transcend static genomic information, transcriptome-guided approaches offer unprecedented resolution for refined diagnosis, definition of a prognosis based on cellular status, and personalized therapeutic selection, which can in itself overcome the challenges of tumor unpredictability and contribute to the next generation of personalized cancer therapies.
